# Long-term health-enhancing physical activity in rheumatoid arthritis - the PARA 2010 study

**DOI:** 10.1186/1471-2458-12-397

**Published:** 2012-07-12

**Authors:** Birgitta Nordgren, Cecilia Fridén, Ingrid Demmelmaier, Gunnar Bergström, Christina H Opava

**Affiliations:** 1 Division of Physiotherapy, Department of Neurobiology Care Sciences and Society, Karolinska Institutet, 23100, Huddinge, SE 14183, Sweden; 2 Institute of Environmental Medicine, Karolinska Institutet, Stockholm, SE 17177, Sweden; 3 Department of Rheumatology, Karolinska University Hospital, Stockholm, SE 17176, Sweden

**Keywords:** Arthritis, Behavior change, Exercise therapy, Social Cognitive Theory, Intervention study, Longitudinal study, Muscle function, Perceived health, Resistance training

## Abstract

**Background:**

People with rheumatoid arthritis (RA) suffer increased risk of disability andpremature mortality. Health-enhancing physical activity (HEPA) could be one importantfactor to reduce this risk. Rising health care costs call for the development and evaluation ofnew modes of rehabilitation, including physical activity in settings outside the health caresystem.

**Methods/Design:**

This cohort study targets 450 patients with RA that do not currently meet HEPA recommendations, recruited from six hospitals reporting to the Swedish Rheumatology Quality Registers (SRQ). We have developed a two-year real-life intervention program including a minimum of twice-weekly circuit training, moderately intense physical activity the remaining days of the week and group meetings to support behavior change every other week. Our hypothesis is that increased physical activity and exercise will improve perceived health, reduce pain and fatigue, increase muscle function and aerobic capacity, impact psychosocial factors and prevent future cardiovascular events. Research questions regard outcomes, retention rates, dose–response matters and the exploration of responder characteristics. This protocol outlines recruitment procedure, design, assessment methods and the intervention program of the study.

**Discussion:**

The PARA 2010 project is designed to expand the knowledge on HEPA in RA by a progressive approach regarding population, setting, intervention, time frames and outcome measures. To our knowledge this is the first long-term HEPA program based on Social Cognitive Theory, and performed in a real life environment to demonstrate if this new setting can promote increased and maintained physical activity in people with RA.

**Trial registration number:**

ISRCTN25539102

## Background

Rheumatoid arthritis (RA) is a chronic disease with major impact on functioning and health. Reduced body functions, particularly aerobic work capacity and muscle function, are common [1]. Increased risk of comorbidity and early mortality, mainly due to cardiovascular disease, are also present [2,3] and seem to be related to the burden of inflammation [4,5] and possibly also to physical inactivity [6]. Physical activity can prevent such risks in the general population [7].

Physical activity is defined as any bodily movements produced by skeletal muscles resulting in energy expenditure [8]. Exercise, a subcategory of physical activity, is characterized as planned, structured and repetitive activities with the objective to improve or maintain aerobic capacity or muscular strength [8]. Guidelines on physical activity to improve and maintain health, here defined as Health-enhancing Physical Activity (HEPA), have recently been updated to include not only 30 minutes of moderately intense physical activity five times a week, but also twice-weekly muscle strength training [9,10]. The recommended moderately intense physical activity five times a week could be replaced with 20 minutes of vigorously intense activities three times a week.

A majority of patients with RA do not accumulate enough HEPA [11,12] and thus, considering the risks and the barriers connected with the disease, HEPA needs to be promoted in this subpopulation. While the safety and benefit of planned and structured exercise in clinical settings are good [13-15] the outcome of HEPA interventions among patients with RA have only been studied in two randomized controlled trials with somewhat contradictory results [16,17]. None of the studies included strength training that is part of the present HEPA guidelines and calls for other settings and strategies. To our knowledge there are also no clinical trials that have investigated HEPA effects on cardiovascular morbidity and mortality in RA. Strategies to implement the adoption of HEPA and its maintenance over time have been poorly described in previous studies on physical activity in RA. Theoretical frameworks [18,19] are useful and readiness to change [19], outcome expectations, self-efficacy and self- regulatory techniques are essential concepts [18]. Physical activity in patients with RA is predicted by perceived benefits, exercise self-efficacy [20-22], fatigue and perceived barriers to exercise [23]. Social support and previous exercise behavior are associated with exercise maintenance [24]. Providers’ effective support of behavior change is crucial in the implementation of new behaviors among their patients. Knowledge about HEPA in RA, attitudes in line with HEPA recommendations and sufficient skills to support behavior change are thus necessary among physiotherapists in rheumatology as well as their ability to guide specific, individualized goal-setting, planning and self-monitoring, give feed-back on performance and guide in strategies to prevent relapse [25,26]. Better developed strategies to implement not only the adoption of HEPA behavior, but also its maintenance over time will probably result in better long-term outcome of HEPA interventions [27].

Matters relating to the identification of responders according to predefined outcomes versus non-responders to HEPA interventions as well as those on dose–response have, to our knowledge, never been addressed in clinical trials of HEPA among patients with RA.

### Aim

The aim of this protocol is to describe the recruitment procedure, design, assessment methods and the intervention program of a HEPA intervention study targeting people with RA. The description of the intervention adheres to the checklist of the TREND statement for nonrandomized evaluations of behavioral and public health interventions [28].

## Design and methods

### Main aim

The main objective of this clinical trial is to study the implementation and outcome of a twoyear HEPA intervention program, based on Social Cognitive Theory, among people with RA that do not currently meet the HEPA recommendations.

#### Specific aims

·To describe perceived health, pain and fatigue, muscle function, aerobic capacity, activity limitation, disease activity, HEPA and psychosocial factors, as well as their inverse relationships among those who volunteer to participate in a HEPA program.

·To investigate the outcome of a two-year HEPA program on perceived health, pain and fatigue, muscle function, aerobic capacity, HEPA, psychosocial factors and cardiovascular events.

·To investigate retention rates and differences between completers and non-completers.

·To explore HEPA dose–response issues and identify responder characteristics.

#### Hypothesis

Our main hypothesis is that the intervention program will increase HEPA and consequently improve perceived health, reduce pain and fatigue, increase muscle function and aerobic capacity, impact psychosocial factors and prevent future cardiovascular events.

### Design

This clinical trial is a multicenter cohort study with a longitudinal design, which is part of the larger PARA 2010 study. The intervention sample will be compared to a representative comparison sample.

### Participants

#### Inclusion criteria

**Figure 1:**
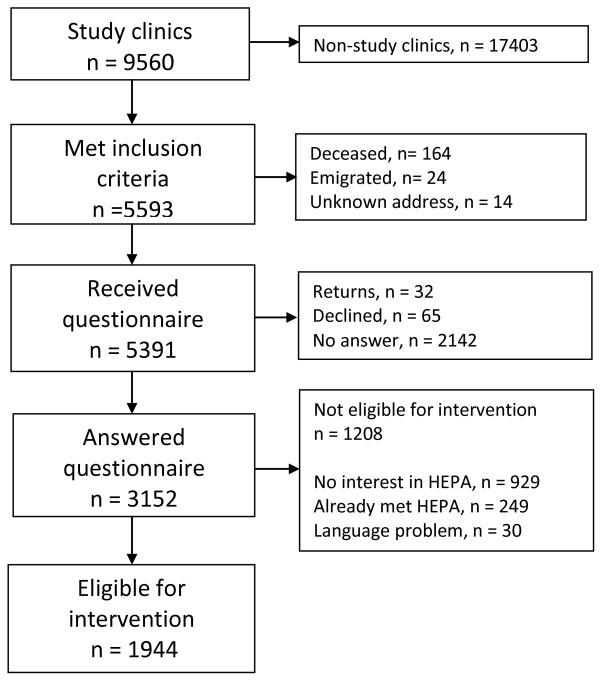
Flow chart of recruitment procedure of the study.

A flow-chart depicting the selection of the study sample is included in Figure 1. All patients with RA according to the ACR criteria [29], up to age 75 years and independent in daily living were identified from six rheumatology clinics, chosen to represent university hospitals and county hospitals in urban and rural areas, reporting to the SRQ. Patients fulfilling the inclusion criteria were mailed a questionnaire and those returning it and fulfilling additional inclusion criteria, i.e. expressing interest in organized exercise, speaking and understanding Swedish without major problems, not already obtaining HEPA, and not having other major diseases preventing HEPA, were asked to participate in the intervention study. Those consenting to participate, showing up for initial assessments and starting the intervention form the intervention sample. Those fulfilling all inclusion criteria, but declining participation for various reasons, form a representative comparison sample.

#### Sample size

A power analysis indicated that 91 participants per group would confer conclusive results

(β = 0.2, α = 0.05) with general health perception (0–100, primary endpoint) as the basis for the analysis. Since the VAS is considered an ordinal scale no attention was paid to the magnitude of the differences in change between groups, but rather to the proportion to the participants that was expected to improve (40 % and 20 % in intervention sample and representative comparison sample respectively) during the intervention. Full-powered gender-separated analyses, considering that only 20-25 % of patients with RA are men, thus require 450 participants in each group.

### Intervention

**Figure 2:**
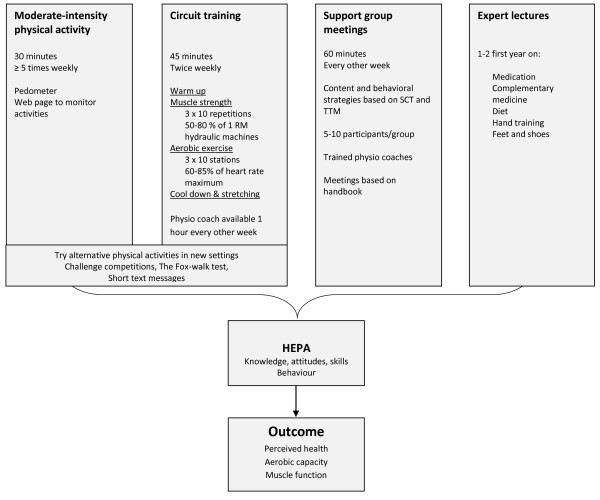
Intervention components.

For description of intervention components see Figure 2.

#### Moderate-intensity physical activity

During the first study year, each participant is encouraged to perform moderate-intensity physical activity at least 30 minutes on most days of the week. They are introduced to and provided with a pedometer and free access to a web page for registration and monitoring of their physical activity [30].

#### Circuit training

Participants are also encouraged to take part in at least two weekly 45-minute circuit training sessions. The circuit consists of 20 stations; ten of which provide muscle strength training of eight major muscle groups and the other ten provide aerobic exercises. The equipment is hydraulic and produces concentric resistance relative to exercise speed. Each station takes 30 seconds and three circuit laps result in 3x10 repetitions of each task [31]. A physical therapist is initially present to instruct and assist in adjusting the program to each participant’s needs and preferences. The participants commit to pay the costs related to circuit training at a public gym during the first year of the project.

#### Strategies for maintenance, relaps prevention and performance feed-back

Alternative types of HEPA, individually or together with group peers, are encouraged. To prevent relapse during holidays, challenge competitions are organized where participants report their HEPA and may win simple prizes, e.g. towels. Furthermore, the participants are encouraged to perform the Fox-walk test, a simple method to monitor aerobic capacity by walking a track [32] and they are also provided with short message service (SMS), weekly text messages to monitor and encourage their HEPA.

#### Support group meetings

**Figure 3:**
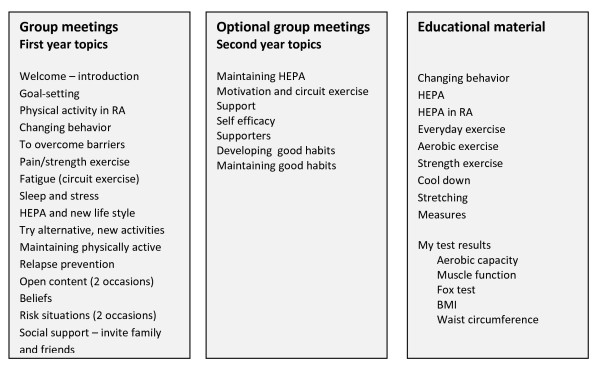
Content of the handbook for use by participants at the support group meetings.

During the first year, physical therapists guide support group meetings with 5–10 participants one hour every other week, to facilitate learning of specific behavioral skills to enable incorporation of exercise sessions and moderate-intensity HEPA into daily routines. A study-specific handbook (Figure 3) is used, and the following behavioral strategies are incorporated: (i) specific and individual goal-setting that is systematically evaluated and adjusted, (ii) selfmonitoring of progress towards set goals, (iii) mutual feedback on performance, (iv) problemsolving to help overcome present and future barriers, and (v) relapse-prevention. Each group meeting includes the above elements and, in addition, discussions on a specific topic such as ‘HEPA and RA’, ‘Pain and strength training’, ‘Sleep and stress’, or ‘HEPA and the environment’. The handbook also includes general information on behavior change, HEPA, aerobic exercise, muscle strength training and the performance tests used in the study. During the group sessions, knowledge, attitudes and self-efficacy for HEPA based on the participants’ previous experiences are discussed and integrated in the individual goal-setting. The group format enables social support, positive reinforcement of HEPA and observational learning by sharing experiences with other participants. During the second year, monthly group meetings are optional with the participants in charge.

#### Expert lectures

Expert lectures on participants’ preferred topics, e.g. medication, diet and complementary medicine, are offered once or twice during the first year.

### Training of 'phsyio coaches'

In order to address treatment integrity [33-35], physical therapists experienced in rheumatology ('physio coaches') are trained to deliver the intervention. The training comprises a number of elements.

Six joint course days, including two two-day sessions before the intervention and two one-day booster sessions during the first year of the intervention, are provided. The course includes knowledge acquisition about HEPA in RA and on strategies to support HEPA behavior. The main focus is on learning coaching skills to support the strategies described for group meetings above. Role play and self-selected home assignments are used to practice challenging coaching situations. Study specific treatment protocols are presented, clearly stating which core components should be included in the coaching during group meetings in the early, middle and late phases of the first intervention year. A written manual, based on the content of the participant handbooks and describing the content of the group meetings, is also presented to physio coaches. The outline of the group meetings, although with fixed core components, allows for adjustments according to the specific needs of each group.

On-site visits are made by one of the researchers (BN) to the physio coaches’ local gyms to instruct and discuss correct performance of the circuit training in order for the participants to obtain enough exercise intensity and load. Each coach is also introduced to, and provided with a heart rate monitor enabling them to give feed-back on their participants’ performance.

The physio coaches are video recorded and given feed-back on behavioral performance by one of the researchers (ID) at two selected group meetings during the first year. Sequences from the video recordings, selected to serve as good examples of coaching and enabling observational learning among the coaches, is published on an internet community.

### Assessments

All participants in the intervention sample and the representative comparison sample are assessed at baseline and after one year and two years with data retrieved from the SRQ, patient files and a questionnaire. In addition, the intervention sample is assessed with performance tests and anthropometric measures. Assessments are presented in Table 1 and described in detail below.

**Table 1:** Assessments performed during the intervention

**Assessments**	**Baseline**	**One year**	**Two years**
**Data retrieved from SRQ**^**1**^**and patient files**			
DAS 28^2^	X	X	X
Data on medication	X	X	X
Data on cardiovascular events		X	X
Demographics, language-skills, co-morbidity	X	X	X
General health perception, VAS^3^	X	X	X
Pain, VAS	X	X	X
Fatigue, VAS	X	X	X
EQ5D^4^	X	X	X
HAQ^5^	X	X	X
IPAQ^6^	X	X	X
ESES^7^	X	X	X
mFABQ^8^	X	X	X
ESAI^9^	X	X	X
SSEB^10^	X	X	X
Outcome expectations	X	X	X
TST^11^	X	X	X
Grippit	X	X	X
Aerobic capacity	X	X	X
**Anthropometry and blood pressure (intervention participants only)**			
Waist circumference	X	X	X
BMI^12^	X	X	X
Blood pressure	X	X	X
**Questionnaire** Opinions on intervention		X	
**Questionnaire** HEPA^13^ maintenance			X

^1^ Swedish Rheumatology Quality Registers,^2^ Disease Activity Score,^3^ Visual analogue scale,^4^ EuroQol,^5^ Stanford Health Assessment Questionnaire, ^6^ International Physical Activity Questionnaire, ^7^ Exercise Self-efficacy Scale, ^8^ modified Fear-avoidance Beliefs Questionnaire, ^9^ Exercise Stage Assessment Instrument, ^10^ Scales to measure social support for exercise behaviors, ^11^ Timed stands test, ^12^ Body mass index, ^13^ Healthenhancing physical activity.

#### Assessors

To assure the quality of assessments, physiotherapists, other than those coaching the intervention, from all participating clinics are initially trained to administer the questionnaires, perform the physical performance tests and calibrate the test equipment in a standardized setting. This training is performed under the supervision of an experienced physiotherapist during four days before the start of the intervention and then again before the one-year and two-year follow-ups.

#### Data retrieved from the SRQ and patient files

- Disease Activity Score (DAS28) measures inflammatory activity, based on erythrocyte sedimentation rate, number of swollen and tender joints and self-reported general health perception (VAS 0–100 mm scale) [36]. The DAS28 is scored 0–10 with scores below 3.2 indicating low disease activity and those above 5.1 as high.

- Data on medication classified as disease-modifying anti-rheumatic drugs, oral steroids and biologics.

- Cardiovascular events, i.e. transitory ischemic attack, myocardial infarction, hypertension, congestive heart failure and stroke.

#### Data retrieved from questionnaires

- Sociodemographic characteristics; income, education, language comprehension and family situation.

- Comorbidity; chronic obstructive pulmonary disease, asthma, emphysema, stroke, myocardial infarction, hypertension, neurologic or psychiatric disease.

- General health perception rated on a 100 mm visual analogue scale (VAS) from ‘Totally fine’ (=0) to ‘Worst imaginable health’ (= 100), which is considered valid and reliable in RA [37].

- Perceived pain rated on a 100 mm VAS from ‘No pain’ (= 0) to ’Maximal pain’ (= 100), which is considered valid and reliable in RA [38].

- Fatigue rated on a 100 mm VAS from ’No fatigue’ (= 0) to ’Maximal fatigue’ (= 100). The fatigue VAS has good face validity and is sensitive to change in RA [39,40].

- Health outcome with the EuroQol (EQ 5D 3 L), a standardized questionnaire consisting of two parts [41]. The EQ 5D 3 L descriptive systems comprise 5 dimensions: mobility, self-care, usual activities, pain/discomfort and anxiety/depression. Each dimension is scored from ‘No problems’ (= 1) to ‘Extreme problems’ (= 3) Furthermore, to rate health the actual day, a line is drawn from a box to the appropriate point on a vertical thermometer from ‘Worst imaginable health state’ (= 0) to ’Best imaginable health state’ (= 100). EQ 5D 3 L has construct validity in RA, is responsive to change and is satisfactorily reliable for group comparisons [42].

- Activity limitation with the Stanford Health Assessment Questionnaire (HAQ) [43]. It comprises 20 questions and is scored on four levels from ’With no difficulty’ (= 0) to ’Unable to perform’ (= 3) addressing activities of daily living performed within the past week: dressing and grooming, arising, eating, performing hygiene, reaching, gripping, walking and common daily activities. The HAQ is valid and reliable in RA [44,45].

- Health-enhancing physical activity with the International Physical Activity Questionnaire (IPAQ) short version, a self-administered questionnaire collecting information about physical activity at several intensity levels and across several domains (home, work, transport and leisure time), undertaken over the past seven days before the assessment. The short version has acceptable test-retest reliability and criterion-related validity compared with accelerometers in the general population [46].

- Exercise self-efficacy measured with the Exercise Self-efficacy Scale (ESES) [47,48] including six items about exercising despite various barriers. Responses are given on11-point numerical rating scales from ’Not at all confident’ (= 0) to ’Very confident’ (= 10). Satisfactory internal consistency and test-retest reliability have previously been reported in college students [47] and in patients with musculoskeletal pain [49].

Beliefs about physical activity causing pain and injury with the modified Fear-avoidance Beliefs Questionnaire (mFABQ) [50]. Four items are responded to a 7 point scale with ‘Do not agree at all’ (= 0) and ‘Agree completely’ (= 7) as anchors. mFABQ has previously been used in the general population and in patients with RA [50,51].

- Exercise stage of change with the Exercise Stage Assessment Instrument (ESAI) [52], modified to fit the purpose of the present study. Response options range from ’Yes, I have been for more than 6 months’ to ’No, and I do not intend to the next 6 months’. The questionnaire has sufficient construct validity compared to other exercise stage of change measures [53].

- Social support for HEPA from family and friends with the Scales to measure social support for exercise behaviors (SSEB) [54] consisting of two 13-item scales assessing the frequency of different types of support for HEPA from family and friends respectively during the previous three months using six point scales from ‘Does not apply’ (= 0) to ‘Very often’ (= 5). The SSEB has satisfactory internal consistency and test-retest reliability [54].

- Outcome expectations for physical activity measured with two self-reported questions: ‘How certain are you that HEPA is beneficial for your health in the long run?’ and ’How certain are you that HEPA has a positive impact on your RA-related difficulties?’. The response format is 10-grade numerical rating scales from ’Not at all sure’ (= 1) to ’Totally sure’ (= 10) [55].

#### Performance tests

- Lower limb function with the Timed Stands Test (TST), measuring the time in seconds required for ten full stands from a sitting position in an armless chair [56]. The TST is valid and reliable in patients with RA [57]. Norm value for healthy men and women aged 20–85 years are available [56].

- Grip strength with the Grippit (AB Detektor, Göteborg Sweden), an electronic dynamometer that measures maximum momentary force (peak force) in Newtons (N), mean force during ten seconds sustained grip and force for the final 0.5 seconds [58]. After a first warm-up trial, the participant is asked to squeeze the dynamometer as hard as possible for ten seconds without verbal encouragement given by the examiner. The mean of three trials is used for analyses [59]. The Grippit is reliable in people with RA [58]. Norm values for healthy men and women aged 20–69 years are available [58].

- Maximal aerobic capacity estimated from a submaximal bicycle ergometer test and expressed as maximal oxygen consumption (VO_2_max) in l/min and ml x min x kg^-1^ respectively. Fitness values are classified from ‘low’ to ‘high’ in order to enable comparison across individuals, independent of sex and age. The test is performed according to Åstrand & Rhyming [60] and a predicted VO_2_value is obtained on a given work load, the observed heart rate (HR) and the participants’ weight, age and sex. A 15-grade rating scale of perceived exertion (RPE) is used for subjective rating of exertion [61]. Participants on beta blockers perform the test, but only the perceived exertion is registered since HR is not reliable [62]. The same test equipment is used at each clinic at baseline and follow-ups, and participants are tested in a standardized setting [60]. HR is registered with a Polar watch F6 (Polar Electro Oy, Kempele, Finland).

#### Anthropometric measures

- Waist circumference measured just below the lower costal rib using non extensible measuring tape [63].

- Body mass index (BMI) calculated by dividing body weight in kilograms by the square of body height in meters.

- Blood pressure tested before the bicycle ergometer test with a sphygmomanometer and a stethoscope.

#### Adherence to HEPA

- SMS with cellular phones, an ecological momentary assessment (EMA) that permits the participant to report HEPA close in time to experience, is used to monitor adherence [64]. Two text messages are sent automatically at the same time every week with a reminder two days later: “How many times during the past week have you performed the circuit training?. Please answer by pressing a number between 0-7” and “In addition to the circuit training performed, how many of the remaining days during the past week have you been physically active for at least 30 minutes on a moderate level? Please answer by pressing a number between 0-7”. Answers are automatically transferred to a data file and stored under safe conditions. SMS have previously been used in preventive medicine and in promoting health behaviors but not in relation to HEPA adherence [65-67]. The reported response rate in a population treated for low back pain was 82.5 % and the method was found to be user- friendly [68].

#### *Questionnaires on opinions about intervention and HEPA maintenance - first year*

-Study specific questions on different elements of the intervention during the first year with the opportunity to make personal comments include: support group attendance, use of weekly planning, HEPA performance, use of pedometer and step registration at web site, answering SMS, use of the Fox-walk test, and participation in challenge competitions and expert lectures. They are asked to rate the value of physical therapist and peer support, handbooks, weekly planning, circuit training, physical activity in daily life, pedometer and web page registration, SMS, heart rate monitoring, Fox-walk test, challenges and expert lectures from ‘Not at all valuable’ (=1) to ‘Very valuable’ (=5). They are also asked whether they would recommend a relative or a friend with similar symptoms to participate in a program for physical activity with the same approach, with five answering options from ‘No definitely not’ to ‘Yes, definitely’.

#### Questionnaires on opinions on intervention and HEPA maintenance - second year

-Study specific questions on different elements of the intervention during the second year with the opportunity to make personal comments include: support group attendance (number, organization, content, contact modes, use of handbooks, use of weekly planning, HEPA performance, use of pedometer and step registration at web site, answering SMS, use of the Fox-walk test. They are asked to rate the value of peer support at group meetings, peer support for HEPA, handbooks, weekly planning, circuit training/alternative training, physical activity in daily life, pedometer and web page registration, SMS and Fox-walk test from ‘Not at all valuable’ (=1) to ‘Very valuable’ (=5).

#### Treatment integrity

Treatment integrity is assessed by the video recordings and by logs kept by the physiocoaches including: (i) the content of each group meeting, (ii) attending participants, (iii) any adverse events and (iv) structured self-reports of own coaching behavior in relation to the predefined core components.

### Planned data analyses

Non-parametric statistics will mainly be used in cross-sectional and longitudinal analyses due to the ordinal data produced by most assessment methods used. Unpaired and paired tests, correlation coefficients as well as ANOVAs will be used to analyze within-group and between-group differences and changes. In addition, logistic regression models and/or cluster analyses will be performed to analyze issues related to dose–response and responder characteristics.

## Discussion

The current project is designed to expand the knowledge on HEPA in RA by a progressive approach regarding population, setting, intervention, time frames and outcome measures. It integrates physiological and behavioral aspects of HEPA behavior, aiming to assess the impact on self-perceived general health.

A majority of earlier studies have been performed within the health care system in clinical settings under supervision of physiotherapists. We aim to identify a subgroup within the RA population motivated to increase their physical activity to HEPA levels, likely to manage their HEPA in settings outside the health care organization. The setting for this study is public gyms with easy-to-use equipment, attracting a varied population with and without activity limitations. To our knowledge this is the first long-term HEPA program performed in a real life environment to demonstrate if increased and maintained physical activity in people with RA can be promoted in this new setting. With limited resources of health care, groups capable of self-management should be identified, thus freeing resources for patients in need of more support and care.

Our choice of recruitment via the SRQ for a matched cohort study with one intervention sample confer excellent opportunities to perform thorough analyses to characterize those consenting to participate compared to those declining, those adopting HEPA compared to non-adopters, and those reporting beneficial health outcomes compared to those who do not benefit, thus enabling conclusions about different subgroups in an RA population. The comparison sample constitutes the rest of our total target sample, can be monitored via the SRQ and is similar to the intervention sample as regards age, activity limitation and interest in a HEPA intervention. Although our focus is on the intervention sample, we expect the representative comparison sample to mirror the natural course of RA during the study period. Our belief is that this design fits our purposes better than a randomized controlled design that may jeopardize long-term compliance with the HEPA intervention as well as with the control conditions.

The intervention is based on physiological and behavioral knowledge of HEPA in RA. We emphasize the intensity and load of everyday activity and exercise, and use theory on behavioral change to guide the content of the intervention as well as the training of the physiocoaches. The theoretical framework used in this study, the Social Cognitive Theory, is in line with directions for future research on how to design, deliver and evaluate self-managementprogram in patients with RA [69]. The design of the intervention corresponds well with a recent review of physical activity trials concluding that interventions are more likely to achieve maintenance if they are conducted over more than 24 weeks, include some face-to face contact, use more than six behavioral strategies and include follow-up prompts [70]. The use of weekly SMS reports as a measure of HEPA and a cue for performing HEPA behavior has not been evaluated in an RA population previously. Our study uses a long-term approach by performing the intervention in two steps during two years; the group intervention is performed during the first year and the participants are responsible for the continuation of HEPA during the second year. The rationale is that individuals need time to initiate, adopt and maintain new behaviors in order to reduce the risk of relapsing into previous unwanted behaviors.

Our intervention is evaluated from different perspectives and thus a multitude of assessment methods are used. While most questionnaires in our study are valid for use either in the general population or for people with musculoskeletal conditions, the validity of the answers could still be questioned due to an extensive amount of questions. However, since the questionnaires are mailed, the participants may choose to fill them in over a couple of days and thus reduce the burden and the threat to the validity of their answers.

We expect that our HEPA program, integrating physiological and behavioral aspects of HEPA, will encourage and support the participants to reach the new guidelines on HEPA and thus improve general health perception, reduce pain and fatigue, increase muscle function and aerobic capacity, and have beneficial long-term effects on cardiovascular events. The results are expected to be externally valid for a subgroup within the RA population, motivated to manage their own HEPA behavior in settings outside the health care system. Results from this intervention study are expected to be published from 2013.

## Ethics

Ethics approval has been obtained from the Stockholm regional research ethics committee (2010/1232-31/1). All survey and cohort participants will be asked for participation by a letter containing information about the study. Those willing to participate will be given the opportunity for additional information through telephone calls.

## Competing interests

The authors declare that they have no competing interests.

## Authors’ contributions

CO is responsible for identification of the overall research questions, design of the study, acquisition of ethics approval and funding, participant recruitment, supervision of the study realization, and participated in the drafting of this manuscript. BN contributes with compilation of questionnaires and protocols for performance assessments, the production of participant handbooks, planning and execution of independent assessor training, and prepared the initial draft of this manuscript. CF contributes with compilation of protocols for performance assessments, production of participant handbooks, planning and execution of independent assessor training, and participated in drafting this manuscript. ID is responsible for planning and execution of the education of the physiocoaches, production of participant handbooks, and participated in drafting this manuscript. GB contributes with planning and execution of the education of the phsyiocoaches and critically reviewed this manuscript. All authors approved the final manuscript.

## Pre-publication history

The pre-publication history for this paper can be accessed here:

http://www.biomedcentral.com/1471-2458/12/397/prepub
